# Confluent and Reticulated Papillomatosis Successfully Treated with Topical Vitamin A Derivative

**DOI:** 10.1155/2023/9467084

**Published:** 2023-03-06

**Authors:** Manal Alsulami, Bader Alharbi, Yaser Alotaibi, Fadi Alghamdi, Adel Alsantali

**Affiliations:** ^1^College of Medicine, King Abdulaziz University, Jeddah, Saudi Arabia; ^2^Department of Dermatology, King Fahad Armed Forces Hospital, Jeddah, Saudi Arabia

## Abstract

Confluent and reticulated papillomatosis (CARP) is a rare dermatosis that typically develops in adolescents and young adults. Clinical characteristics include hyperkeratotic papules that coalesce centrally with a reticulated pattern peripherally on the central and upper trunk, neck, and axilla. Its etiology is not precisely known, and disordered keratinization has been postulated as one of the etiologies. Treatment options of the disease include systemic (such as antibiotics, antifungals, and retinoids) and topical treatments (such as lactic acid, antifungals, retinoids, salicylic acid, urea, tacrolimus, and vitamin D analogs). We report a case of a 17-year-old boy, otherwise healthy, presented with a new onset of asymptomatic, persistent, and slowly progressing brownish skin lesions over the trunk for 6 months. The diagnosis was revised to CARP based on clinical and histopathological examination. Treatment with topical tretinoin 0.025% cream once daily was begun. There was complete resolution of his lesions at the end of 8 weeks of therapy. There has been no relapse at 2 months follow-up. The effectiveness of tretinoin in this patient supports the theory that CARP is a keratinization disorder. Initiating treatment with topical tretinoin when no limitations for its use would be reasonable as it can provide a safer alternative to systemic therapy.

## 1. Introduction

Confluent and reticulated papillomatosis (CARP) is a rare dermatosis originally described by Gougerot and Carteaud and also called Gougerot–Carteaud syndrome [1]. It is characterized by hyperkeratotic or verrucous grey-brownish papules that coalesce into confluent plaques centrally with a reticulated pattern peripherally [2]. It is commonly distributed on the central and upper trunk, neck, and axilla in adolescents and young adults [3]. The diagnosis is largely made on a clinical basis but the role of histopathology remains an important measure to exclude other dermatoses. Its etiology remains uncertain, which explains the diversity of therapy options with variable outcomes [4]. Treatment options for the disease include systemic and topical treatments. Systemic treatment includes antibiotics (such as minocycline, doxycycline, tetracycline, and azithromycin), antifungals, and retinoids (isotretinoin and acitretin). Topical treatment includes lactic acid, antifungals, retinoids, salicylic acid, urea, tacrolimus, and vitamin D analogs [1]. Herein, we describe a case of CARP that was successfully treated with topical tretinoin.

## 2. Case Report

**Figure 1:**
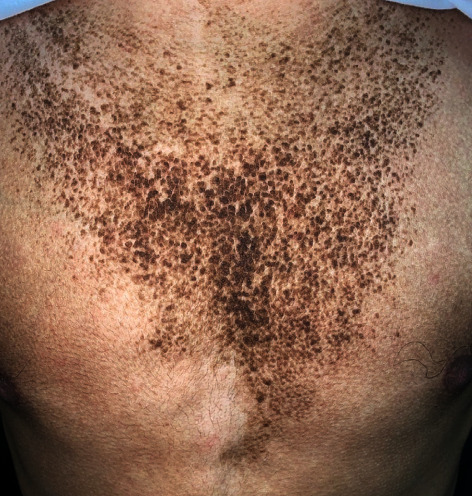
Hyperpigmented, hyperkeratotic, confluent macules, and papules that coalesce into reticulated plaques over the upper trunk.

**Figure 2:**
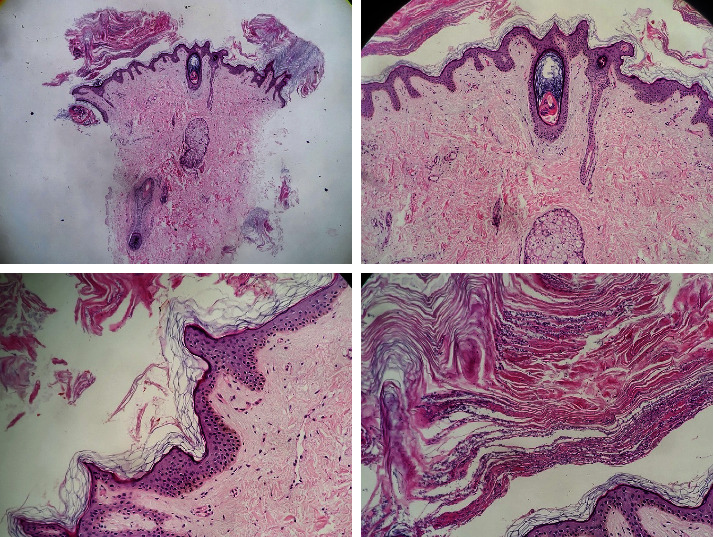
A skin biopsy showing marked hyperkeratosis with papillomatosis in the epidermis and numerous fungal organisms (yeasts) in the corneal layer. A very scant chronic perivascular inflammatory infiltrate was noted in the dermis with edema and fibrosis.

**Figure 3:**
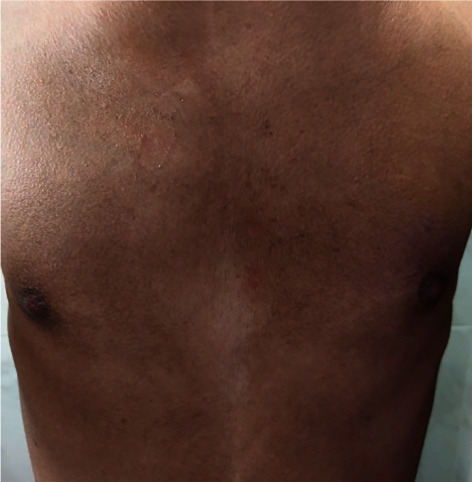
Resolution of the lesion within eight weeks of treatment.

**Figure 4:**
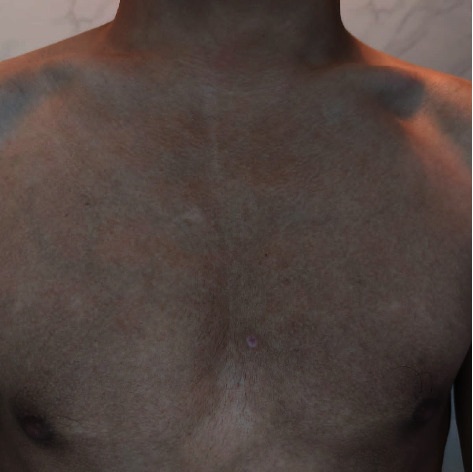
No recurrence at two-month follow-up.

A 17-year-old boy, otherwise healthy, presented with a new onset of asymptomatic, persistent, and slowly progressing brownish skin lesions over the trunk for 6 months. Past medical history, drug history, and family background were noncontributory. Examination revealed hyperpigmented, hyperkeratotic, confluent macules, and papules that coalesce into reticulated plaques over the upper trunk (Figure 1). No fluorescence was observed under a Wood's light examination. Rubbing the lesion with 70% isopropyl alcohol was unrevealing. Oral and nail examinations were unremarkable. A skin biopsy showed marked hyperkeratosis with papillomatosis in the epidermis and numerous fungal organisms (yeasts) in the corneal layer. A very scant chronic perivascular inflammatory infiltrate was noted in the dermis with edema and fibrosis (Figure 2). Based on the clinical and histopathological findings, a diagnosis of CARP was made. The patient was unwilling to start oral minocycline. Therefore, we initiated treatment with topical tretinoin 0.025% cream once daily. In 5 weeks, the patient started to notice a marked improvement with the flattening and fading of the papules and plaques. Within 8 weeks, the condition was almost completely resolved (Figure 3). No recurrence was noted at 2 months follow-up (Figure 4).

## 3. Discussion

CARP is a rare skin disorder that preferentially affects young adults without particular sex predilection [3]. Its pathogenesis is not precisely known, but there are various theories. It was thought to be caused by *Malassezia furfur* as CARP clinically resembles tinea versicolor. Still, studies have not been consistent with the detection of yeasts in patients with CARP. The current infectious theory is that CARP is caused by the bacteria, *Dietzia papillomatosis*. Disordered keratinization has been postulated as an etiology that is either familial or acquired [1]. Reports of cases that were successfully treated with vitamin A derivatives support this theory [5–7]. Other etiologies include diabetes, obesity, ultraviolet light, and amyloidosis [1].

In regard to the diagnosis of CARP, Davis et al. proposed the following criteria: (i) clinical findings include scaling brown macules and patches, at least part of which appear reticulated and papillomatosis; (ii) involvement of the upper trunk and neck; (iii) fungal staining of scales is negative for fungus; (iii) fungal staining of scales is negative for fungus; (iv) no response to antifungal treatment; and (v) excellent response to minocycline [8].

Histopathologically, the most common findings are hyperkeratosis, papillomatosis, and acanthosis. The dermis may contain perivascular lymphocytic infiltrates, mild dilatation of superficial dermal blood vessels, beading of elastic fibers, and hypermelanosis of the basal layer [9]. Our case was compatible with these typical features of CARP.

In our case, we considered tinea versicolor, terra firma-forme dermatosis, Dowling–Degos disease, and Darier disease as differential diagnoses. Tinea versicolor was unlikely considering the absence of fluorescence on Wood's light examination. We swabbed the lesion with 70% isopropyl alcohol but it did not remove it, thus ruling out terra firma-forme dermatosis. Dowling–Degos disease was not favored as it usually presents as reticulated hyperpigmentation without papules or plaques and presents with a family history of similar lesions. Darier disease was ruled out due to the absence of oral, nail, palmoplantar changes, and the lack of suprabasal acantholysis and dyskeratotic cells in histopathology, in addition to the absence of family history [4, 10].

Numerous treatment modalities have been used for CARP. Antibiotics such as minocycline, which was considered the first-line treatment, were reported to yield good results. Despite the successful response seen in patients treated with minocycline, relapse is common after discontinuation, and long-term treatment with antibiotics has its associated risks [8]. Doxycycline, tetracycline, and azithromycin are three other antibiotics that have been shown to be effective in treating some patients. The effectiveness of these antibiotics is mainly attributed to their anti-inflammatory properties [4].

Topical selenium sulphide was found to be beneficial in a variety of cases [11, 12]. It has both keratolytic and antifungal properties, and its effect could be due to its keratolytic effect rather than its antifungal properties [12].

Topical (tretinoin) and oral (isotretinoin and etretinate) vitamin A derivatives have also been used with success [5–7, 13, 14], whereas other studies have not shown improvement with topical tretinoin treatment [13, 14]. Other topical therapies that have been used successfully include lactic acid, urea, salicylic acid, calcipotriol, and tacrolimus [1].

Our patient responded very well to topical tretinoin (0.025% cream once daily). Continuing the treatment for a longer period is recommended to see if there is a possibility to taper the medication without causing a relapse of CARP. The major limitation of using topical tretinoin is that its application is cumbersome when extensive areas are involved and areas hard to reach in self-application (such as the back) are affected. Therefore, if a patient is able to apply the medication as the lesion is localized and easy to reach in self-application, similar to our case, topical tretinoin may provide a safer alternative to systemic therapy.

## 4. Conclusion

CARP is an uncommon and curable dermatosis. The effectiveness of tretinoin in this patient supports the theory that CARP is a keratinization disorder. Initiation of treatment with topical tretinoin when there are no limitations on its use would be reasonable, as it can provide a safer alternative to systemic therapy.

## Data Availability

The data used to support the findings of this study are included within the article.
